# Infant feeding practices and risk of preschool obesity in AlAin, UAE: A cross-sectional study

**DOI:** 10.1371/journal.pgph.0002803

**Published:** 2024-02-08

**Authors:** Dana AlTarrah, Julie Lanigan, Jack Feehan, Ayesha S. Al Dhaheri, Syed M. Shah, Leila Cheikh Ismail, Atul Singhal

**Affiliations:** 1 Department of Social and Behavioral Sciences, Faculty of Public Health, Kuwait University, Kuwait City, Kuwait; 2 Joint institution UCL Great Ormond Street Hospital, Institute of Child Health, University College London, London, United Kingdom; 3 Institute for Health and Sport, Victoria University, Melbourne, Australia; 4 Department of Nutrition and Health, College of Medicine and Health Sciences, United Arab Emirates University, Al Ain, United Arab Emirates; 5 Institute of Public Health, College of Medicine and Health Sciences, United Arab Emirates University, Al Ain, United Arab Emirates; 6 Department of Clinical Nutrition and Dietetics, College of Health Sciences, University of Sharjah, Sharjah, United Arab Emirates; 7 Department of Women’s and Reproductive Health, University of Oxford, Nuffield, Oxford, United Kingdom; VART Consulting PVT LTD, INDIA

## Abstract

Early childhood obesity is serious public health problem, and poses a risk of obesity in later life. The study aimed to investigate whether infant feeding affects risk of overweight and obesity in preschool children in the United Arab Emirates (UAE). A cross-sectional study was carried out. Data was collected in a kindergarten in Al Ain, UAE. One hundred and fifty parents and preschool children aged 2 to 6 years participated in the study. Univariate and multivariate linear regression were used to investigate associations. A longer duration of breastfeeding and later introduction of complementary foods were associated with a lower BMI z-score in preschool children. Each month of any breastfeeding was associated with a lower BMI z-score in the unadjusted model (β = -0.03; 95% CI -0.05, -0.01; p = 0.01), and each month increase in the age of introducing complementary foods was associated with a lower BMI z-score in the unadjusted model (β = -0.43; 95% CI: -0.60 to—0.027; p<0.001). These associations remained after adjustment for potential confounding factors (age, sex, maternal BMI, maternal education level, mother’s age, social class, father’s BMI) for duration of breastfeedinig (β = -0.02; 95% CI: -0.05 to 0.00; p<0.001) and age of complementary feeding (β = -0.39; 95% CI: -0.57 to—0.21; p<0.001). Poor infant feeding practices (shorter duration of breastfeedinig and early introduction of complementary foods) were found to be associated with higher BMI in preschool children. Promoting appropriate proper infant feeding practices in line with recommendations could be one strategy to help prevent childhood obesity in the UAE.

## Introduction

Childhood overweight and obesity is a critical public health challenge of the 21^st^ century [1]. The prevalence of childhood overweight and obesity is high, particularly in children under the age of five [2]. Obesity has become a particular problem in transitional countries, which have undergone dramatic socio-demographic and economic changes in the past decades including the oil-producing Arab Gulf countries where rapid urbanisation and the adoption of a ‘Western’ lifestyle have been suggested to be contributory factors [3–5]^.^ However, while the prevalence of overweight and obesity and its risk factors are well documented in school-aged children, adolescents and adults [6–11], studies focused on preschool children are scarce.

Multiple risk factors, starting from early life, have been suggested to influence a young child’s risk of overweight and obesity [3, 12, 13]. These include: genetic, environmental, developmental, behavioural and dietary factors [14, 15]. However, most studies have been carried out in developed countries and little is known about the influence of these risk factors in the Arabian Gulf region. Identification of modifiable risk factors of preschool obesity is important, in order to develop appropriate and effective interventions to prevent obesity. This study aimed to identify risk factors of preschool overweight and obesity in a Kindergarten in Al Ain, in the United Arab Emirates (UAE), focusing on infant feeding practices in early life.

## Materials and methods

### Ethics statement

This study was conducted according to the guidelines laid down in the Declaration of Helsinki and all procedures involving research human subjects. Ethical approved was attained from the National Research Ethics Committee at the College of Medicine and Health Sciences, United Arab Emirates (Project No. 14/37), and the Research Ethics Committee at University College London both approved the study protocol (Project ID: 5618/001). Written informed consent was obtained from all subjects before the commencement of the study.

The study was conducted from October 2^nd^ to November 30^th^, 2014 in children aged between 2 and 6 years old attending the Emirates National School in Al Ain, UAE. All 402 children attending the school were eligible to participate in the study except those with a pre-existing clinically diagnosed medical condition. After fully explaining the study, information booklets were sent to all parents and informed written consent was obtained. Four hundred and two parents of children attending the school were contacted to participate in a single blind randomized controlled trial (RCT) [16], of which a random sample of 150 parents and children agreed to participate. Baseline data from the RCT were used in the study.

### Sample size calculation

The cross-sectional study was part of a randomised controlled trial that investigated the effectiveness of a healthy lifestyle tool to prevent preschool overweight and obesity [16]. Sample size calculations were based on a previous study of an obesity intervention [17] with one hundred and forty-four school-aged children were required to detect a 0.5 standard deviation difference in BMI z-score (assuming 5% significance and 80% power) in groups randomised to an intervention designed to prevent obesity or to control

### Anthropometry

Anthropometric measurements were carried out using standard protocols. Body weight was measured using electronic scales accurate to 0.1kg (Seca, CMS weight equipment Ltd., London, UK), and height was measured using a portable stadiometer (Leicester Height Measure MK 11, London, UK). Weight and height were used to calculate Body Mass Index (BMI) as weight/height^2^. BMI standard deviation scores (z-scores) were calculated using LMS software in Microsoft Excel [18]. Children below 5 years were classified as overweight where BMI z-score was at or above +2SD and obesity as a BMI z-score at or above +3SD using the WHO 2006 growth standard. For children over 5 years of age, the WHO 2007 reference was used, and overweight and obesity were defined where BMI z-scores were at or above +1SD and +2SD respectively.

### Socio-demographic measures

The educational status of parents was categorised according to the mother’s educational attainment (with university degree, without university degree). In the UAE, there is no distinct categorisation of social class according to the National Bureau of Statistics (2011) and was therefore was determined based on the occupation of the father, thus a dichotomous division (manual, non-manual) was used to allow comparisons with previous research. Data on parents’ marital status (married, divorced), maternal ethnicity (Emirati, non-Emirati), mother’s age (years), father’s age (years), and smoking practices (including all types such as smoking cigarettes, ‘*shisha’* (hookah) and *‘medwakh’* (a smoking pipe) and duration of smoking) were collected by questionnaire.

### Parental overweight and obesity

Self-reported height and weight of parents was collected by questionnaire, and BMI was calculated from these measurements. Overweight was classified as BMI between 25 and 29.99 kg/m2 and obesity was classified by BMI above 30 kg/m2 [19].

### Early life factors

Data on pregnancy, birth weight and infant feeding practices were collected from mothers by questionnaire [20]. Maternal recall using questionnaires is reported to be a valid and reliable estimate of infant feeding practices, especially when the duration of breastfeeding is recalled within the preschool years [21]. Mothers were asked whether the child had been breastfed or not, the age when they completely stopped being fed breast milk, the age when they were first fed formula, the age when they completely stopped drinking formula, and the age when they were introduced to solids (complementary feeding). Responses were used to calculate the duration of exclusive or any breastfeeding, and the duration of formula feeding, in months. Although most analyses were based on continuous data, the data was further categorised for dichotomous analysis. Birth weight was categorised as low (<2.5kg), normal (2.5kg-3.9kg), and high (>4.0kg), while breastfeeding was categorised into ever breastfed (yes, no), any formula feeding(yes, no), exclusively breastfed for 4 months or 6 months (yes, no), duration of any breastfeeding (never, less than 2 months, 3–5 months, more than 6 months), and age of complementary feeding (before or after 4 months).

### Statistical analysis

Data were analysed using SPSS for Windows (version 24.0; IBM, NY). Descriptive statistics were used to report mean and standard deviations for continuous data, and frequency statistics were used to calculate numbers and percentages for categorical variables. Distributions of continuous data were inspected using Q-Q plots and histograms and assessed for normality with the Kolmogorov-Smirnov test. Data that were not normally distributed were log_e_ transformed and multiplied by 100 prior to analysis. The mean of log_e_ transformed data represents a geometric mean and the standard deviation of the 100 log_e_ transformed data represents the coefficient variation [18]. Independent t-tests were used to compare continuous variables and chi-square was used for categorical variables.

Univariate linear regression was used to investigate associations between socio-demographic variables, infant feeding practices and BMI z-score. Variables that reached significance or presented a notable trend in the univariate analysis were included as covariates in multivariate analyses. A full model was developed where risk factors (socio-demographic, parental and infant feeding practices) and potential confounding factors (age, sex, mother’s BMI, father’s BMI, maternal educational level, social class and mother’s age) were entered simultaneously. Confounding variables included in the model were selected based on existing knowledge of confounders influencing obesity risk factors, and findings of previous analyses. A beta coefficient with 95% Confidence Intervals (CI) was calculated to identify variables associated with BMI z-score. Statistical significance was set at p < 0.05 for all analyses.

## Results

The characteristics of the study population are summarised in **Table 1**. Of the 150 preschool children included, 57% were boys, and the mean age of children was 4.4 ± 0.6 years. Most of the study population were Emirati nationals 144 (96%). Seventeen children (11%) children were classified as overweight/obese, and no gender differences were found in rates of obesity/overweight. Overweight/obesity rates were closely comparable to the prevalence reported in a recent large study by AlBlooshi et al., (2016). (n = 6,731), where 753.9 (11.2%) of children aged between 3 and 6 years were overweight and obese (BMI for age >95th percentile) [22].

**Table 1:** Sociodemographic, parental, pregnancy and infant feeding practices.

	All n = 150		Girls n = 65		Boys n = 85		p^1^
**Age, years**	4.4	(0.8)	4.4	(0.8)	4.4	(0.8)	0.8
**Mother**							
Education: with degree, n (%) ^2^^, 3^	82	(56)	32	(49)	50	(59)	0.2
Ethnicity: Emirati nationals, n (%) ^2^	144	(96)	64	(99)	80	(94)	0.2
Marital status: married, n (%) ^2^	145	(97)	63	(97)	82	(97)	0.9
Mother age, y ^3^	32.9	(4.8)	32.6	(4.9)	33.1	(4.8)	0.5
Mother BMI, kg/m^2^ ^3^	27.6	(5.0)	27.8	(5.4)	27.4	(4.6)	0.6
**Paternal**							
Social class: Manual, n (%) ^2,^ ^3^	5	(3)	1	(2)	4	(5)	0.4
Father age, y ^4^	37.0	(6.7)	36.1	(5.7)	37.6	(7.4)	0.2
Father BMI, kg/m^2^ ^4^	28.5	(5.0)	28.3	(4.7)	28.8	(5.1)	0.7
Father smoking, n (%) ^2^^,^ ^5^	38	(25)	19	(29)	19	(22)	0.4
**Anthropometry**							
Height, cm^1^	104.9	(6.9)	103.8	(7.0)	105.7	(6.7)	0.1
Weight, kg^1^	17.1	(3.2)	17.1	(3.6)	17.2	(2.8)	0.8
BMI, kg/m^2^ ^1^	15.5	(1.7)	15.7	(2.0)	15.3	(1.3)	0.1
BMI z-score^1^	0.01	(1.1)	0.2	(1.3)	-0.1	(1.0)	0.2
Weight status^2^							
Not overweight/obese, n (%)	133	(89)	55	(85)	87	(92)	0.2
Overweight/Obese, n (%)	17	(11)	10	(15)	7	(8)	
**Pregnancy characteristics**							
Parity	3.7	(1.7)	3.9	(1.7)	3.7	(1.7)	0.4
Birth weight, kg	2.9	(0.5)	2.8	(0.6)	3.0	(0.5)	0.2
**Infant feeding Practices**							
Duration exclusive breastfeeding, months^1^	3.0	(2.3)	3.2	(2.2)	2.9	(2.4)	0.5
***Exclusive breastfeeding*^*2*^**							0.5
Never, n (%)	40	(27)	15	(23)	25	(29)	
<2 months, n (%)	26	(17)	10	(15)	16	(19)	
3–6 months, n (%)	84	(56)	40	(62)	44	(52)	
***Exclusive breast feeding*^*2*^**							1.0
< 4 months, n (%)	76	(51)	33	(51)	43	(48)	
> 4 months, n (%)	74	(49)	32	(49)	42	(49)	
***Exclusive breast feeding*^*2*^**							1.0
< 6 months, n (%)	116	(77)	50	(77)	66	(78)	
> 6 months, n (%)	34	(23)	15	(23)	19	(22)	
Duration of any breastfeeding, months^1^	11.0	(8.0)	10.6	(8.3)	11.2	(7.8)	0.6
***Duration of any breastfeeding*^*2*^**							0.8
Never breastfed, n (%)	4	(3)	2	(3)	2	(2)	
Ever breastfed, n (%)	146	(97)	63	(97)	83	(98)	
***Duration of any breastfeeding*^*2*^**							0.6
Never breastfed, n (%)	4	(3)	2	(3)	2	(2)	
< 6 months, n (%)	65	(43)	31	(48)	34	(40)	
> 6 months, n (%)	81	(54)	32	(49)	49	(58)	
Duration of formula feeding, months^1^	23.5	(13.0)	22.7	(13.3)	24.1	(12.6)	0.5
Age of complementary feeding	4.9	(1.0)	4.8	(1.1)	5.0	(1.0)	0.1
Complementary feeding							
< 4 months, n (%)	14	(9)	10	(15)	4	(5)	**0.03**
> 4 months, n (%)	136	(91)	55	(85)	81	(95)	

All data mean (SD) unless indicated. Significance p<0.05

^1^ Comparison between groups using independent t-test, except

^2^ Comparison between dichotomous variables using chi-squared test

^3^ < 3% missing data

^4^ <15% missing data

^5^ smoking was only reported by fathers.

The mean duration of exclusive breastfeeding or any breastfeeding was 3 months and 11 months respectively. Forty three percent were breastfed for less than 6 months and 27% were never exclusively breastfed, while 56% were exclusively breastfed between 3 and 6 months. The average age of complementary feeding was 4.9 months, and 91% of children were introduced to solids after 4 months **(Table 1).**

Duration of breastfeeding and age of complementary feeding were associated with BMI z-score. Each month of any breastfeeding was associated with a lower BMI z-score in the unadjusted model (β = -0.03; 95% CI: -0.05 to -0.01; p = 0.01). This effect was attenuated after adjustment for confounders (age, sex, mother’s BMI, age, and education level, father’s BMI and social class). Each one month increase in the duration of any breastfeeding was associated with -0.02 lower BMI z-score (95% CI: -0.05 to 0.00; p = 0.05) (**Table 2**). This association remained unchanged following the addition of birth weight to the model (β = -0.02; 95% CI: -0.05 to 0.00; p = 0.05).

**Table 2:** Association of socio-demographic, parental, birth weight and infant feeding practices with BMI z-score.

	Unadjusted^1^			Adjusted^2^		
	β	(95% CI)	p	β	(95% CI)	p
Age, years^3^	-0.11	(-0.33, 0.12)	0.3	0.03	(-0.21, 0.27)	0.8
Gender: Male^3^	0.27	(-0.10, 0.64)	0.2	0.30	(-0.09, 0.70)	0.1
**Maternal**						
Mother’s age, years^3^	-0.04	(-0.08, -0.002)	**0.04**	-0.04	(-0.09, 0.00)	**0.05**
Mother’s BMI, kg/m^2^ ^3^	0.03	(-0.01, 0.06)	0.2	0.03	(-0.01, 0.07)	0.1
Educational level: with degree, n (%)^3^	0.32	(-0.05, 0.69)	0.1	0.34	(-0.06, 0.75)	0.1
Parity	-0.09	(-0.20, 0.02)	0.1	-0.07	(-0.23, 0.09)	0.4
Ethnicity: Emirati, n (%)^3^	0.20	(-0.74, 1.14)	0.4	0.21	(-0.81, 1.23)	0.7
Marital status: Married, n (%)^3^	-0.72	(-1.74, 0.30)	0.2	0.61	(-1.59, 2.83)	0.5
**Paternal**						
Father’s age, years	-0.01	(-0.04, 0.01)	0.3	0.01	(-0.03, 0.05)	0.7
Father’s BMI, kg/m^2^ ^3^	0.04	(-0.00, 0.08)	0.1	0.03	(-0.01, 0.07)	0.2
Social class: non-manual, n (%)^3^	0.94	(-0.07, 1.94)	0.1	0.74	(-0.26, 1.75)	0.1
**Pregnancy and infant feeding**						
Birth weight, kg	0.40	(0.05, 0.75)	**0.03**	0.34	(-0.05, 0.72)	0.1
Duration of exclusive breastfeeding, months	-0.06	(-0.14, 0.02)	0.2	-0.05	(-0.14, 0.03)	0.2
Duration of any breastfeeding, months	-0.03	(-0.05, -0.01)	**0.01**	-0.02	(-0.05, 0.00)	**0.05**
Duration of any formula feeding, months	-0.003	(-0.02, 0.01)	0.7	-0.00	(-0.02, 0.01)	0.7
Age of complementary feeding, months	-0.43	(-0.60, -0.27)	**<0.00111**	-0.39	(-0.57, -0.21)	<**0.001**

^1^ Linear regression analyses; significance set at p<0.05

^2^ Multivariate linear regression analyses adjusted for Age, Sex, Maternal BMI, Maternal Education level, Mother’s age, Social class, Father’s BMI.

^3^ Variable excluded in corresponding adjusted model

No associations were found between the duration of exclusive breastfeeding (p = 0.2) or duration of formula feeding (p = 0.7) and BMI z-score, in either unadjusted or adjusted models (**Table 2**).

Later age of complementary feeding was associated with a lower BMI z-score. BMI z-score was -0.43 lower with each additional month (95% CI -0.60 to -0.027; p<0.001). The association remained after adjustment for confounding factors (each month increase in the age of complementary feeding was associated with a -0.39 lower BMI z-score (95% CI -0.57 to 0.21; p<0.001) and confounding factors together with birth weight (β = -0.38; 95% CI: -0.57 to—0.19; p<0.001) (S1 Table). Following adjustment for confounders, only gender differences for the regression analysis for age of complementary feeding and BMI z-score were found. However, no significant interactions between gender and age of complementary feeding on BMI z-score were identified (S2 Table).

## Discussion

In the present study a longer duration of breastfeeding and a later introduction of complementary foods were found to be strongly associated with lower BMI z-score. These findings are similar to extensive previous research from Western populations and suggest that, as in these countries, promotion of healthier infant feeding practices could help the prevention of early childhood obesity in the UAE.

### Longer duration of breastfeeding and overweight/obesity risk

There is extensive and accumulating evidence which suggests that breastfeeding is protective against childhood obesity [23–25]. For example, a one meta-analysis concluded that breastfeeding was associated with a 26% reduced risk of obesity in childhood and adulthood [25]. In a sub-analysis of the highest quality studies, where adjustment of confounders was included, a 13% risk reduction was found. The size of the association between duration of any breastfeeding and later risk of overweight in the present study (each month of any breastfeeding was associated with a lower -0.03 BMI z-score (CI: -0.05 to -0.01) was similar to previous research from Western populations. However, despite the extensive epidemiological evidence linking breastfeeding and later obesity this causal association remains highly debated [13]. Overall, although impossible to prove causality, there is a consensus that breastfeeding is likely to be protective against later obesity [25–27]. However, no previous study has investigated the association between breastfeeding and preschool obesity in the UAE.

Several previous studies have reported that infant feeding practices are suboptimal in the UAE and neighbouring Arab Gulf countries [28–30]. In the present study, although breastfeeding rates were high (97% of children were ever breastfed, with only 3% exclusively formula fed) and similar to those reported by a another study in the UAE, where 95% of Emirati women breastfed from birth [28], the duration of exclusive breast-feeding was low (exclusive breastfeeding at 6 months only 23%). By 2025 the WHO aims to achieve a 50% universal exclusive breastfeeding rate [31], however the low rates of exclusive breastfeeding found in the present study are similar to those reported in the UAE [28, 31]. While the current study did not qualitatively investigate reasons for the low rate of exclusive breastfeeding, previous studies have suggested that early supplementation with formula milk and early introduction of non-milk fluids and foods (aniseed drinks, gripe water, tea, sweetened water with dates) are considered a norm in the UAE, as mothers believe breast milk alone is insufficient [28, 29].

Interestingly, although global initiatives have focused their efforts on promoting exclusive breastfeeding for maternal and child health benefits, evidence for an association between exclusive breastfeeding, as opposed to any breastfeeding, and childhood obesity is inconsistent [13, 23, 27]. For instance, in the current study, exclusivity of breastfeeding was not found to be associated with BMI z-score. Although some studies have found an inverse association between exclusive breastfeeding and obesity risk [32–35], there is currently no consensus that exclusive breastfeeding, irrespective of its duration, is protective against obesity or high BMI in later childhood [27]. Discrepancies cited in the literature could be due to differences in definitions of breastfeeding exclusivity, or the influence of other unmeasured behavioural factors in previous studies. However, as the present study was relatively underpowered, it was not possible to evaluate categorical associations (i.e. less than 4 or 6 months) or provide robust evidence for this association.

Several biological and behavioural mechanisms have been suggested to explain the association between duration of breastfeeding and lower risk of later childhood obesity. The lower protein content of breast milk compared to formula milk has been suggested to help prevent fast growth during infancy [36] and therefore reduce the risk of obesity in later life. In addition, the ability of breastfed infants to self-regulate their intake in response to internal satiety cues may also be important [32]. Finally, because breast milk provides optimal nutrition for infants up to the age of 6 months [37], delays in timing of introduction of complementary foods may also help reduce the risk of obesity[26, 38, 39]. However, the observational nature of these studies cannot confer causality or exclude the influence of residual confounding.

### Later introduction of complementary foods and overweight/obesity risk

The present study also found strong evidence that later introduction of complementary foods is associated with a lower BMI z-score. These findings are consistent with previous studies linking early introduction of complementary foods and later childhood overweight or obesity [35, 40, 41]. Similar to the present study, findings from the UK Millennium Cohort (n = 13,188) found that early introduction of complementary foods before 4 months was associated with overweight in 3-year-old children, even after adjusting for confounding factors (adjusted OR = 1.12; 95% CI 1.02 to 1.23) [42]. Furthermore, a recent meta-analysis found that the introduction of complementary foods before four months of age, compared with 4 to 6 months was associated with an increased risk of overweight and obesity in 5 out of 8 studies (pooled Relative Risk (RR) = 1.18; 95% CI: 1.06 to 1.31) [43].

Importantly, heterogeneity between studies, the observational nature of investigations, and the errors related to retrospective recall of infant feeding data need to be taken into consideration when interpreting findings, however, the current study in the UAE is consistent with most of the literature in Western countries [43–46], and suggests that the early introduction of solids, prior to 4 months, could be one risk factor for later childhood overweight and obesity.

The mechanism behind early introduction of complementary foods and higher risk of overweight or obesity remains unclear [13]. Potential explanations include higher energy and/or protein intake with complementary feeding which could increase insulin-like growth factor-1 concentrations and promote faster infant weight gain and later adiposity in childhood [40, 45]. Another potential mechanism is that early introduction of complementary foods may displace breastfeeding, and therefore reduce the protective effects of breastfeeding against childhood obesity [26]. Several reasons have been suggested to explain why mothers choose to introduce solids. These include the perception that breast milk is insufficient, the lack of family support, or, in some cases, mothers may opt to use traditional remedies to soothe the infant, such as diluting dates in water or formula milk [28, 30].

Interestinlgy, several putative risk factors for preschool overweight and obesity described western popultions were not found in the current study. These include high maternal BMI, low maternal educational level, low social class, in sufficient physical activity level, more time spent in sedentary behaviours, and sub-optimal sleep duration (S3 Table) [12, 44, 47]. Possible resaons for this include the small study sample size or possible cultural differences between the UAE and western countries. For example, while the risk of obesity is found to be highest in low socioeconomic groups in Western countries, obesity seems to be a problem of the affluent (high socioeconomic groups) in middle income countries [4, 48–50]. However, little is known about how this relationship operates in the UAE, which has undergone a nutrition transition. While, a positive trend was observed between indicators of high socio-economic status (mothers’ high levels of education and fathers’ nonmanual occupations) and children’s BMI z-score, similar to trends observed in other developing countries [50, 51]. This association in countries in transition could be explained by greater access to energy-dense foods, electronic devices (e.g. iPads, TV sets), and fast food restaurants, which may in turn lead to a positive energy balance [5, 52]. Moreover it could possibly be due to the few mothers of low socio-econmic status included in the study.

### Strengths and limitations

The present study faced a number of limitations. Firstly, the small sample size recruited at one study location may have introduced bias, with underpowered statistical analyses and reduced generalisability of the study findings. Secondly, the observational nature of the study cannot determine causality. Thirdly, the present study population may not be representative of the UAE population. However, the ratio of Emiratis to non-Emiratis in the kindergarten chosen for the current study was comparable to a recent study in carried out in 91 kindergartens in Ras Al Khaimah (UAE), where over 90% of students were Emirati citizens [22]. Nevertheless, the study investigated several putative risk factors (sociodemographic, birth weight, behavioural factors) of preschool obesity. Additionally, objective anthropometric measurements were carried out by trained researchers. Moreover, considering the scarcity of data on preschool children in the region, this study highlights the need for prospective cohort studies with a larger representative sample size to strengthen the findings.

## Conclusion

The current study supports the hypothesis that infant feeding practices in the first year for life may influence the risk of later obesity in the UAE. This association may be particularly important in the UAE, where there is poor adherence to infant feeding recommendations. Collectively, these findings suggest that early preventative strategies should focus on mothers in order to promote appropriate infant feeding practices. Furthermore, considering the protectiveness of breastfeeding against childhood obesity, public health efforts and nutrition policies to promote breastfeeding need to be strengthened. Future longitudinal studies and intervention trials are needed to further inform infant feeding guidelines and improve existing breastfeeding promotion campaigns.

## Supporting information

S1 Checklist Inclusivity in global research. (DOCX)

S1 Table Association of birth weight and infant feeding practices with BMI z-score. (DOCX)

S2 Table Association of infant feeding practices with BMI z-score. (DOCX)

S3 Table Association of socio-demographic, parental, and behavioural factors with BMI z-score. (DOCX)

S1 Data Excel files raw data. (XLSX)
