# Supplementary material for: Infant feeding practices and risk of preschool obesity in AlAin, UAE: A cross-sectional study
Source: PLOS Glob Public Health. 2024 Feb 8;4(2):e0002803. doi: 10.1371/journal.pgph.0002803 (PMC10852324; doi:10.1371/journal.pgph.0002803)
Supplement: S1 Table — (DOCX) [file pgph.0002803.s002.docx]

|  | Unadjusted^1^  β (95% CI) | p | Adjusted^2^  β (95% CI) | p | Adjusted^3^  β (95% CI) | p |
| --- | --- | --- | --- | --- | --- | --- |
| Birth weight, kg | 0.40 (0.05, 0.75) | **0.03** | 0.34 (-0.05, 0.72) | 0.1 | - | - |
| Duration of exclusive breastfeeding, months | -0.06 (-0.14, 0.02) | 0.2 | -0.05 (-0.14, 0.03) | 0.2 | -0.05 (-0.14, 0.03) | 0.2 |
| Exclusive breastfeeding |  |  |  |  |  |  |
| < 4 months^4^ | 1.00 (ref) | 0.1 | 1.00 (ref) | 0.3 | 1.00 (ref) | 0.3 |
| > 4 months | -0.32 (-0.68, 0.05) |  | -0.22 (-0.61, 0.17) |  | -0.22 (-0.61, 0.17) |  |
| Duration of any breastfeeding, months | -0.03 (-0.05, -0.01) | **0.01** | -0.02 (-0.05, 0.00) | **0.05** | -0.024 (-0.05, 0.00) | **0.05** |
| Breastfeeding |  |  |  |  |  |  |
| Never breastfed^4^ | 1.00 (ref) | 0.9 | 1.00 (ref) | 1.0 | 1.00 (ref) | 1.0 |
| Ever breastfed | -0.09 (-1.23, 1.06) |  | -0.02 (-1.14, 1.09) |  | 0.01 (-1.09, 1.12) |  |
| Duration of any formula feeding months | -0.003 (-0.02, 0.01) | 0.7 | -0.00 (-0.02, 0.01) | 0.7 | -0.01 (-0.02, 0.01) | 0.5 |
| Age of complementary feeding, months | -0.43 (-0.60, -0.27) | **<0.001** | -0.39 (-0.57, -0.21) | <**0.001** | -0.38 (-0.57, -0.19) | **<0.001** |
| Age of complementary feeding |  |  |  |  |  |  |
| < 4 Months^4^ | 1.00 (ref) | **<0.001** | 1.00 (ref) | **<0.001** | 1.00 (ref) | **<0.001** |
| > 4 months | -1.84 (-2.40, -1.29) |  | -1.48 (-2.14, -0.83) |  | -1.46 (-2.12, -0.79) |  |
| ^1^ Univariate linear regression analyses, significance p<0.05  ^2^ Multivariate linear regression analyses adjusted for Age, Sex, Maternal BMI, Maternal Education level, Mother’s age, Social class, Father’s BMI  ^3^ Multivariate linear regression analyses adjusted for Age, Sex, Maternal BMI, Maternal Education level, Mother’s age, Social class, Father’s BMI, Birth weight  ^4^ Comparison against reference group in analyses | | | | | | |

S1 Table Association of birth weight and infant feeding practices with BMI z-score
