# Supplementary material for: Infant feeding practices and risk of preschool obesity in AlAin, UAE: A cross-sectional study
Source: PLOS Glob Public Health. 2024 Feb 8;4(2):e0002803. doi: 10.1371/journal.pgph.0002803 (PMC10852324; doi:10.1371/journal.pgph.0002803)
Supplement: S2 Table — (DOCX) [file pgph.0002803.s003.docx]

S2 Table Association of infant feeding practices with BMI z-score

|  | | Unadjusted^1^ |  | Adjusted^2^ |  |
| --- | --- | --- | --- | --- | --- |
|  |  | β (95% CI) | p | β (95% CI) | p |
| Duration of exclusive breastfeeding, months | Boys | -0.02 (-0.11, 0.07) | 0.7 | -0.02 (-0.12, 0.08) | 0.7 |
|  | Girls | -0.12 (-0.26, 0.02) | 0.1 | -0.09 (-0.25, 0.07) | 0.3 |
|  |  |  |  |  |  |
| Duration of any breastfeeding, months | Boys | -0.04 (-0.06, -0.01) | **0.01** | -0.03 (-0.06, 0.00) | 0.1 |
|  | Girls | -0.02 (-0.06, 0.02) | 0.3 | -0.01 (-0.06, 0.03) | 0.5 |
|  |  |  |  |  |  |
| Duration of any formula feeding, months | Boys | 0.004 (-0.01, 0.02) | 0.7 | -0.003 (-0.02, 0.02) | 0.8 |
|  | Girls | -0.10 (-0.03, 0.02) | 0.4 | -0.01 (-0.04, 0.02) | 0.5 |
|  |  |  |  |  |  |
| Age of complementary feeding, months | Boys | -0.30 (-0.52, -0.09) | **0.01** | -0.30 (-0.53, -0.07) | **0.01** |
|  | Girls | -0.54 (-0.80, -0.28) | **<0.001** | -0.42 (-0.74, -0.11) | **0.01** |
| ^1^ Univariate linear regression analyses  ^2^ Multivariate linear regression analyses adjusted for Age, Sex, Maternal BMI, Maternal Education level, Mother’s age, Social class, Father’s BMI  Significance p<0.05 | | | | | |
