# Supplementary material for: Infant feeding practices and risk of preschool obesity in AlAin, UAE: A cross-sectional study
Source: PLOS Glob Public Health. 2024 Feb 8;4(2):e0002803. doi: 10.1371/journal.pgph.0002803 (PMC10852324; doi:10.1371/journal.pgph.0002803)
Supplement: S3 Table — (DOCX) [file pgph.0002803.s004.docx]

S3 Table Association of socio-demographic, parental, and behavioural factors with BMI z-score

|  | Unadjusted^1^ | | | Adjusted^2^ | | |
| --- | --- | --- | --- | --- | --- | --- |
|  | β | (95% CI) | p | β | (95% CI) | p |
| Age, years^3^ | -0.11 | (-0.33, 0.12) | 0.3 | 0.03 | (-0.21, 0.27) | 0.8 |
| Gender: Male^3^ | 0.27 | (-0.10, 0.64) | 0.2 | 0.30 | (-0.09, 0.70) | 0.1 |
| Maternal |  |  |  |  |  |  |
| Mother’s age, years^3^ | -0.04 | (-0.08, -0.002) | **0.04** | -0.04 | (-0.09, 0.00) | **0.05** |
| Mother’s BMI, kg/m^2 3^ | 0.03 | (-0.01, 0.06) | 0.2 | 0.03 | (-0.01, 0.07) | 0.1 |
| Educational level: with degree, n (%)^3^ | 0.32 | (-0.05, 0.69) | 0.1 | 0.34 | (-0.06, 0.75) | 0.1 |
| Parity | -0.09 | (-0.20, 0.02) | 0.1 | -0.07 | (-0.23, 0.09) | 0.4 |
| Ethnicity: Emirati, n (%)^3^ | 0.20 | (-0.74, 1.14) | 0.4 | 0.21 | (-0.81, 1.23) | 0.7 |
| Marital status: Married, n (%)^3^ | -0.72 | (-1.74, 0.30) | 0.2 | 0.61 | (-1.59, 2.83) | 0.5 |
| Paternal |  |  |  |  |  |  |
| Father’s age, years | -0.01 | (-0.04, 0.01) | 0.3 | 0.01 | (-0.03, 0.05) | 0.7 |
| Father’s BMI, kg/m^2 3^ | 0.04 | (-0.00, 0.08) | 0.1 | 0.03 | (-0.01, 0.07) | 0.2 |
| Social class: non manual, n (%)^3^ | 0.94 | (-0.07, 1.94) | 0.1 | 0.74 | (-0.26, 1.75) | 0.1 |
| Behaviour |  |  |  |  |  |  |
| Physical activity, hrs/week | 0.02 | (-0.01, 0.05) | 0.2 | 0.03 | (-0.01, 0.06) | 0.1 |
| Sedentary behaviour, hrs/week | -0.00 | (-0.03, 0.02) | 0.8 | 0.01 | (-0.02, 0.03) | 0.5 |
| Sleep duration, hrs/d | 0.09 | (-0.04, 0.21) | 0.2 | 0.06 | (-0.08, 0.19) | 0.4 |
| ^1^ Linear regression analyses; significance p<0.05  ^2^ Multivariate linear regression analyses adjusted for Age, Sex, Maternal BMI, Maternal Education level, Mother’s age, Social class, Father’s BMI.  ^3^ Variable excluded in corresponding adjusted model | | | | | | |
